# Building Information Modeling Learning Behavior of AEC Undergraduate Students in China

**DOI:** 10.3390/bs12080269

**Published:** 2022-08-05

**Authors:** Panyu Peng, Yibin Ao, Mingyang Li, Yan Wang, Tong Wang, Homa Bahmani

**Affiliations:** 1 College of Environment and Civil Engineering, Chengdu University of Technology, Chengdu 610059, China; 2 College of Management Science, Chengdu University of Technology, Chengdu 610059, China; 3 Department of Engineering Management, Sichuan College of Architectural Technology, Deyang 618014, China; 4 Faculty of Architecture and Built Environment, Delft University of Technology, 2628 CD Delft, The Netherlands

**Keywords:** learning behavior, BIM, influencing factors, UTAUT theoretical model, structural equation modeling

## Abstract

With the popularization and application of Building Information Modeling (BIM), the demand for BIM technical talents in the construction industry is increasing. Exploring college students’ BIM technical learning behavior is of great practical significance to improve education activities. Based on the Unified Theory of Acceptance and Use of Technology (UTAUT), this research adds learning attitude variables to construct a theoretical model of influencing factors of college students’ BIM technology learning behavior. Chinese undergraduate students were asked to complete online questionnaires through peer-to-peer contact with sample colleges and universities. Finally, 1090 valid questionnaires were obtained. The students were sampled from research-oriented, applied research-oriented, application-oriented, and private research-oriented universities in seven regions of China: northeast, north, east, south, central, northwest, and southwest. The structural equation model was used to analyze the sampling data. The results indicate that college students’ BIM learning attitude, performance expectations, and social influence positively and directly impact their learning intention, which indirectly impacts their learning behavior. At the same time, promoting factors and learning intention demonstrate a significant positive and direct impact on learning behavior. Therefore, the following suggestions have been put forward to enhance college students’ learning motivation for BIM technology: increase the popularization of BIM technology in colleges and universities and improve the operation level of full-time BIM teachers. The latter enables colleges and universities to continuously and stably export qualified BIM technical talents to society and the market, resulting in a continuous industry development cycle.

## 1. Introduction

The construction industry has recently adopted Building Information Modeling (BIM). Moreover, research and education on BIM technology have become a prevalent topic for builders and educators in the construction industry [1,2]. Relevant research shows that the popularization and application of BIM technology have undergone a significant transformation in the architecture, engineering, and construction (AEC) industry, which has increased the employment competition of fresh graduates of those majors [3]. Moreover, college students’ mastery of BIM technology can promote the application of BIM technology in the whole construction industry and make the industry more efficient [1]. Such a reinforcing circle should be strengthened to accelerate the industry transformation. Therefore, it is necessary to understand the current situation of BIM technology learning behavior among college students and analyze the significant positive influence path of such behavior so that better education can be provided to stimulate the reinforcing circle.

In the BIM application and education domain, scholars mainly focus on correlating industry, project, and education aspects. For example, Ebrahimi et al. [4] seek the interactions of sustainability and BIM in support of existing buildings. Eadie et al. [5] conducted a survey on the use of BIM in the project life cycle, showing that the BIM industry lacks professional knowledge and training, and relevant BIM educators get more opportunities. Moreover, Tsai et al. [6] developed a new online BIM learning course, focusing on improving the BIM education mode. Jia [7] compared the BIM courses in Chinese and American universities and suggested that the BIM courses in China should be integrated with traditional courses. Meanwhile, Guo et al. [8] explored how to bridge the gaps between BIM education and practice from a competency-based education perspective. In contrast, Ding et al. [9] explore the key factors that affect practitioners’ adoption of BIM. Their study showed that BIM motivation, BIM technical barriers, and BIM ability are the significant factors that affect architects’ adoption of BIM technology. Howard et al. [10] use the Unified Theory of Acceptance and Use of Technology (UTAUT) model to understand the individuals’ perceptions towards working with BIM. The results reveal that performance expectancy does not directly affect behavioral intention of personal participation in BIM, signifying that BIM is perceived as an unrewarded addition to existing work processes.

Reviewing the existing literature, it is found that there is a relative lack of research on the learning behavior of BIM technology from the perspective of individual students. In recent years, exploring BIM education from students’ perspectives has been encouraged. Agirbas [11] pointed out that students’ positive attitude towards using BIM software may improve their learning efficiency of professional architectural knowledge. In addition, through the evaluation of BIM learning achievement in China’s Sixth National BIM Graduation Design Innovation Competition of Colleges and Universities, Ao et al. [1] found seven public factors that affect BIM learning performance: (1) ability of the instructor, (2) school (college) atmosphere, (3) teamwork, (4) individual ability, (5) understanding of BIM industry applications, (6) social environment incentives, and (7) achievement demand. Furthermore, referring to the body of work that exists, this research combines the theory of UTAUT to analyze the influencing factors of college students’ BIM learning intention and behavior and their influencing relationships from the perspective of individual learners, aiming at exploring and developing the path of BIM education.

## 2. Literature Review

### 2.1. Research Status of BIM Technology

In the past few decades, the construction industry has become increasingly interested in using Building Information Modeling (BIM) technologies [12]. BIM is meant for the process of creating and managing information, which enables AEC professionals to effectively plan, design, construct, and manage the whole construction project’s life cycle [13]. The whole life cycle phases are indispensable in the application of BIM, and the lack of one aspect will considerably reduce the effectiveness of BIM use [14]. Therefore, BIM education should not be limited to modeling; students must probe into the BIM workflow [6]. Moreover, most of the research on BIM focuses on its application and implementation [15], ignoring the perceived influence at the personal level [10].

### 2.2. Research Status of BIM Education

Currently, more and more attention is paid to BIM education [16,17]. Chegu Badrinath et al. [18] believe that BIM-educated students can visualize and integrate the project data well, which aligns with the current enterprise talent demand. The research and application of BIM are extensive, but some countries still lack BIM promotion. For instance, a study by Kolarić et al. [19] showed that the application of BIM in Croatia and Slovakia has been deficient. Moreover, it is also believed that the improvement of BIM education will greatly impact improving BIM awareness and acceptance and will encourage those countries to integrate BIM technology into the AEC industry further. Scholars in many countries believe that BIM education needs to be improved through popularization of digitalization and improvement of BIM education that can positively affect contextualized learning and the improvement of BIM awareness and promote the development of BIM technology [19,20,21,22,23,24]. The overwhelming development trend of BIM technology makes colleges and universities pay more attention to cultivating BIM technical talents [25]. BIM-into-extended reality (Virtual Reality, Augmented Reality, Mixed Reality) has laid an excellent theoretical foundation for building a complete BIM education system in colleges and universities [26,27,28,29,30]. However, the training scale of BIM technical talents is far from meeting the needs of industrial development, and BIM education cannot completely support the development of AEC industry [7,31]. Although many colleges and universities have incorporated BIM-related courses into the curriculum [32,33,34], Shi [35] considers that the current BIM education cannot felicitously link BIM with other architectural fields in the curriculum. At the same time, people’s shortage of knowledge of new technology leads to negative attitudes, and the consequences may be far-reaching, which will have a massive impact on technology adoption and industry development [36]. Colleges and universities should realize that students’ perception of BIM technology plays a crucial role in cultivating qualified BIM technical talents.

### 2.3. Unified Theory of Acceptance and Use of Technology Model

With the rapid development of information technology, people’s intention to accept and use new technologies has always been a concern for researchers, who have put forward many theoretical models to predict people’s intentions and behaviors. For example, Fishbein and Ajzen [37] proposed the Theory of Reasoned Action (TRA), claiming that an individual’s attitude towards behavior and subjective norms can influence behavior intention, thus further affecting the actual behavior. When theoretical models are applied to various fields, researchers usually choose suitable structures from many models, mainly ignoring the influence of the unmatched part of the model [38]. Therefore, Venkatesh et al. [38] put forward the Unified Theory of Acceptance and Use of Technology (UTAUT) model, which integrates eight models, namely the Theory of Reasoned Action (TRA), Technology Acceptance Model (TAM), Motivational Model (MM), Theory of Planned Behavior (TPB), Combined TAM, TPB (C-TAM-TPB), Model of PC Utilization (MPCU), Innovation Diffusion Theory(IDT), and Social Cognitive Theory (SCT). Venkatesh tested all the above models and integrated them to form a more refined model, UTAUT, which was widely used by later researchers [39,40,41,42,43,44].

UTAUT identifies four key factors: performance expectancy, effort expectancy, social influence, and facilitating conditions. These four key factors jointly influence the users’ behavioral intention and their behavior.

The behavioral intention is affected by the following variables:

1. Performance expectancy: The degree to which individuals feel that using this technology is helpful to their work or study.
2. Effort expectancy: Individuals feel the degree of effort required to use this technology.
3. Social influence: The degree to which individuals feel influenced by the surrounding groups.
4. The user behavior is affected by the following variables:
  a. Behavioral intention: Individuals’ judgment of the subjective possibility of using or learning this technology.
  b. Facilitating conditions: The degree to which individuals feel supported by the organization when using or learning this technology.

The UTAUT model is widely used in the field of education to study students’ acceptance of a specific technology or system. For example, Mohan et al. [45] and Altalhi [46] used the UTAUT model to analyze the application status of MOOCs in higher education, and the relationship between behavioral intention and behavior is deeply discussed. Chatterjee and Bhattacharjee [47] applied the UTAUT model to explore the application of Artificial Intelligence (AI) in higher education. Alasmari and Zhang [48] investigated college students’ acceptance of mobile learning technology. They discovered that such variables as learning expectancy, effort expectancy, social influence, and characteristics of mobile learning are essential predictors of students’ acceptance of mobile learning technology. At the same time, Sidik and Syafar [49] have drawn a similar conclusion from the research on the factors affecting students’ intention to use mobile learning. The results indicated that performance expectancy, effort expectancy, external influence, and service quality positively influence study intention. Abbad [50] applied the UTAUT model to investigate the students’ use of Moodle, an e-learning system. The findings revealed that performance expectancy and effort expectancy could influence students’ behavioral intention to use Moodle (an E-learning system), and behavioral intention and facilitating conditions can directly influence their user behavior. Noble et al. [51] employed the UTAUT model to show that performance expectancy, effort expectancy, and social influence can significantly affect the use intention of Virtual Reality (VR) learning. As shown, the UTAUT model has been widely used in education research. Therefore, this research applies the mature UTAUT model to explore the influential factors and correlation between college students’ intention to learn BIM technology and their user behaviors, aiming to provide theoretical support for further BIM technology education.

## 3. Materials and Methods

### 3.1. Model Construction and Research Hypothesis

**Figure 1:**
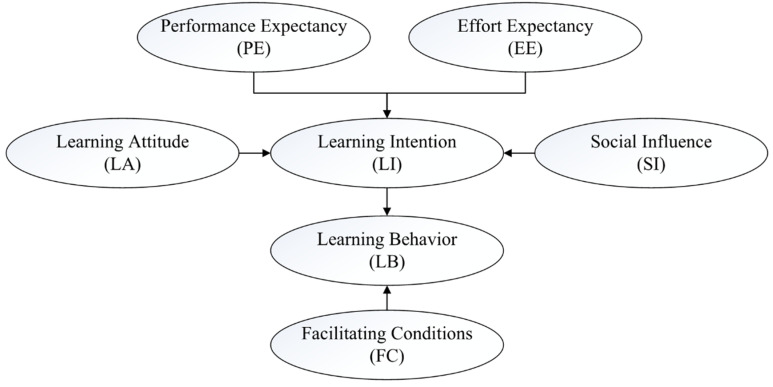
Theoretical framework model of BIM learning behavior research.

This research draws lessons from the existing UTAUT theoretical research results and combines the characteristics of college students, higher education, and BIM technology learning to construct a model of influencing factors of college students’ BIM technology learning intention and behavior. This research model retains the four key factors in the classic UTAUT model: performance expectancy, effort expectancy, social influence and facilitating conditions. “Attitude” is also considered a very strong factor affecting behavioral intention [52,53], so this research considers “attitude” a critical variable in the model. In addition, Dwivedi et al. [54] also mention that most studies using UTAUT often gave up the use of regulatory variables. Arif et al. [55] did not observe the moderating effect of age, gender, and experience in the study of influencing factors on students’ use of distance education based on network services. At the same time, because BIM technology is a compulsory course for AEC majors in most Chinese universities, age, gender, experience, and voluntariness were excluded from the regulated variables. Therefore, the theoretical framework model proposed in this research is shown in Figure 1.

By summarizing the existing research results, this study puts forward the following research hypotheses, as shown in Table 1.

### 3.2. Questionnaire Design

Based on the scale recorded in the existing literature, combined with the BIM learning situation of Chinese AEC majors, this study’s designed questionnaire mainly includes two parts: the first part records the basic information of AEC majors in universities in China, including the interviewees’ gender, age, grade, major, BIM knowledge, and access to BIM courses, and so forth. The second part measures students’ intention and behavior to learn BIM and adopts a Likert five-component scale to measure the degree of each observed variable with 1–5 indicating “totally disagree”, “basically disagree”, “average”, “basically agree”, and “totally agree” respectively. The measurement scale of the questionnaire mainly comes from the UTAUT scale proposed by Venkatesh et al. [38] and the perceived usefulness scale by Davis et al. [52]. Considering the BIM learning context, the learning behavior scale of this study contains 21 items. In addition, a polygraph question is set in the questionnaire: “Please choose the option of completely disagreeing with this question”. If the respondent chooses other options, it is considered that there are some cases, such as choosing directly without reading the questions, and the questionnaire is regarded as invalid. Please see Appendix A for the detailed questionnaire.

### 3.3. Sampling and Date Collection

This study conducted a questionnaire survey in March 2022 using random sampling. The process was divided into three steps: choosing the investigation area, schools, and students.

1. Sampling investigation area: In order to make the research data representative and convincing, the sampled universities cover seven geographical regions in China, including northeast China, north China, east China, south China, central China, northwest China, and southwest China.

2. Sampling colleges and universities: Since BIM technology mainly involves AEC-related majors, universities with civil engineering, engineering management, and other construction majors were mainly selected in the school selection process. According to the types of undergraduate colleges in China, at least one university of four types (research-oriented, applied research-oriented, application-oriented, and private research-oriented) was randomly selected from seven geographical regions to ensure that the sample universities cover the seven regions and four types. We also started point-to-point contact with randomly selected universities to ensure their intention to cooperate. Finally, 35 sample universities were determined, including 8 research-oriented universities, 10 applied research-oriented universities, 8 application-oriented universities, and 9 private research-oriented universities. Sampling information of colleges and universities is shown in Table 2:

3. Sampling students: In this study, online questionnaires were distributed among the students via the teachers from the selected colleges. The research intended to choose at least 30 valid samples in each college, and it was estimated that 1050 valid total samples would be selected.

Data collection mainly went through two stages: the first stage was the pre-investigation stage, which aimed to get feedback to improve the questionnaire quality. This stage started on 20 March and lasted until 26 March 2022. The second stage was the formal investigation stage, which lasted six days, from 27 March to 3 April 2022. The research team contacted AEC-related professional teachers from the selected schools to request they issue online questionnaires. The research team kept tracking and feeding back the questionnaire results, and finally, 1699 questionnaires were collected. A total of 609 invalid questionnaires were eliminated through polygraph questions, and 1090 valid questionnaires were finally recovered with an effectiveness rate of 64.16%. 

Among the 1090 valid questionnaires, 455 were male, accounting for 41.7% of the total and 635 were women, accounting for 58.3% of the total population. The interviewees’ age is mainly between 19 and 22 years old, which is also in line with the age group of undergraduate students. In terms of grades, sophomores account for the most, accounting for 33%; juniors for the next, accounting for 27%; freshmen and seniors for the least, each accounting for 20%. First-year students have just entered the university, so they do not know much about BIM technology and other professional technologies. Hence, their intention to fill out the questionnaire was not firm. Moreover, most seniors have entered internship positions, and the samples collected were few. Table 3 represents the interviewees’ statistics:

## 4. Results and Findings

### 4.1. Reliability and Validity Test

This study used Cronbach’s Alpha coefficient for the reliability test [57]. Generally speaking, Cronbach’s Alpha coefficient should be greater than 0.7 [58]. The Cronbach’s Alpha coefficient of this research data was 0.913, so the reliability of this scale is quite good. In addition, the KMO value of the scale was 0.885, which is greater than 0.7, and the *p*-value in Bartlett Sphericity test is less than 0.05, which is significant. This shows that this scale passed the KMO and Bartlett Sphericity tests and is suitable for factor analysis.

AMOS24.0 software was used to establish the measurement model in this study, and the maximum likelihood estimation method was used for confirmatory factor analysis. The analysis results showed that the convergent validity passed the Average Variance Extracted (AVE) test (AVE > 0.5), and the structure had good convergence [59]. In addition, when the composite reliability (CR) is greater than 0.7, the scale has good convergence validity [60]. The test results of convergent validity of each scale are shown in Table 4. In Table 5, SQRT(AVE) is larger than the correlation coefficients on the horizontal and vertical lines of the table; hence, it shows that the scale has good discriminant validity. To sum up, the reliability and validity of this research scale were tested, and it can be further analyzed.

### 4.2. Goodness of Fit

It can be concluded from the above table that the theoretical model is fitted in this study, and the Chi-square freedom ratio was 4.541, less than 5, which is within the acceptable range. Moreover, RMSEA was 0.057, less than 0.08, and the fitting degree was good. GFI, AGFI, NFI, TLI, and CFI were all greater than 0.9, so the structural equation model in this study has a good fitting degree. Table 6 shows the specific indicators for fitting the research structure model.

### 4.3. Path Analysis 

**Figure 2:**
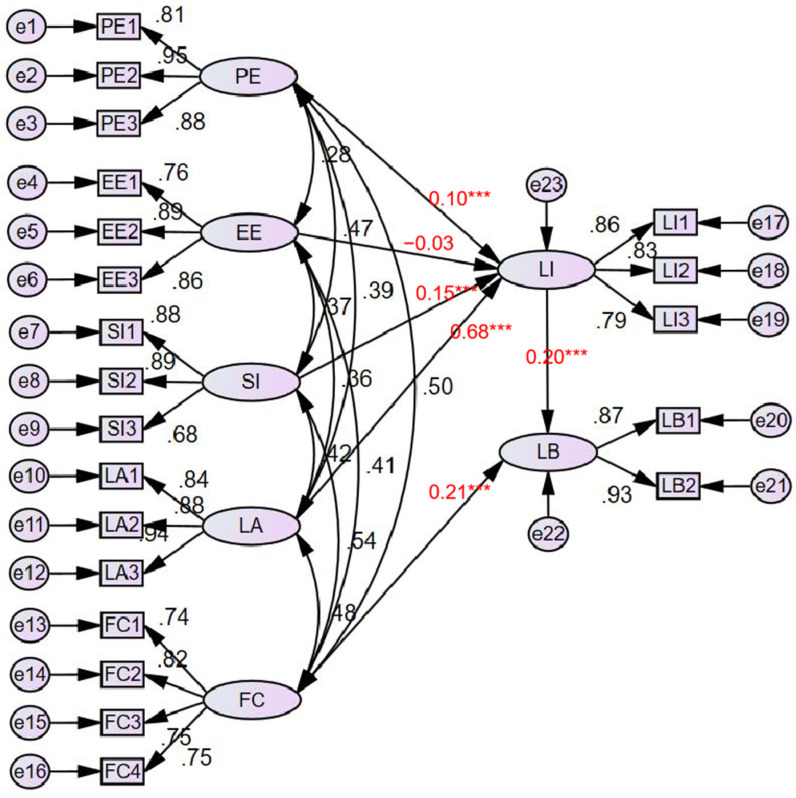
Model analysis diagram of BIM learning behavior research. Note: ** means less than 0.001 < *p* < 0.005, and *** means less than *p* < 0.001.

The fitting results of the model are shown in Figure 2.

It can be seen from Figure 2 that the significance of all paths was less than 0.05, except that efforts expectancy was greater than 0.05, and the standardized regression coefficients were all positive. Among them, the intention to learn BIM was influenced by performance expectancy, social influence, and learning attitude, and the order of influence was learning attitude (0.675) > social influence (0.144) > performance expectancy (0.101). Study behavior was influenced by facilitating conditions (0.212) and behavioral intention (0.203).

In order to explore the influence of exogenous variables on BIM technology learning behavior, this study used “indirect”, “direct”, and “total effects” in the “output” select interface of AMOS24.0 to further analyze the direct, indirect, and total effects of the theoretical model path, and the results are shown in Table 7. Among them, learning attitude, social influence, and performance expectancy significantly indirectly affect learning behavior through learning intention. Compared with social influence and performance expectancy, the indirect effect of learning attitude on learning behavior is more substantial.

## 5. Discussion 

Based on UTAUT theory, this study explores the factors influencing students’ learning behavior toward BIM technology in AEC-related majors. However, it is worth noting that effort expectancy has no significant effect on learning intention.

Firstly, through the verification of BIM technology learning intention and behavior model, we found that learning attitude (β = 0.675, *p* < 0.001) has the greatest predictive effect on college students’ BIM technology learning intention, while learning attitude (β = 0.137, *p* < 0.001) has a significant indirect impact on learning behavior through learning intention. In this study, among the three observed variables that constitute attitudes, LA2 (I am interested in BIM technology) and LA3 (It is fun to learn BIM technology) have the highest factor loads. It can be seen that when college students are more interested in BIM technology, their intention to learn is higher. This finding is similar to the conclusion drawn by Mohan et al. [45], manifesting that students are more willing to learn when they think MOOCs are more attractive.

Furthermore, this study also verified that performance expectancy (β = 0.101, *p* < 0.001) and social influence (β = 0.144, *p* < 0.002) have a significant direct influence on college students’ intention to learn BIM technology. In addition, performance expectancy (β = 0.02, *p* < 0.001) and social influence (β = 0.029, *p* < 0.002) have a considerable indirect influence on learning behavior. If college students think that learning BIM technology is helpful to their studies, job hunting, and career development, they will have a higher learning intention of BIM technology. The higher learning intention will significantly and indirectly influence their BIM technology learning behavior. Research by Choukas-Bradley et al. [61] shows that young people are easily influenced by their peers. For example, when classmates, teachers, and other people who can influence college students’ behavior think they should learn BIM technology, they will also increase their intention to learn. On the other hand, when college students realize that BIM technology is developing well in the construction industry, it will also effectively enhance their intention to learn BIM technology. The industry and educational circles have been working together to improve BIM teaching, integrating BIM education into AEC courses in universities [3]. BIM-related courses are obligatory for AEC majors in many universities [32,33,34]. However, before entering colleges and universities, many students have never experienced working with BIM technology, and most of them are exposed to BIM technology at the request of teachers or the recommendation of senior seniors. Under such conditions, the social influence on students will play a great role.

Moreover, facilitating conditions (β = 0.212, *p* < 0.001) positively impact college students’ BIM technology learning behavior. When the school supports college students in learning BIM technology, they can get help from teachers or classmates when they encounter difficulties learning BIM technology. As a result, their learning can be improved. For example, research by Ao et al. [1] showed that the teacher’s guidance significantly positively impacts students’ BIM learning performance. In addition, students’ learning behavior can be positively influenced by facilitating their access to the required resources from the school library or online resources while learning BIM technology.

However, unlike the conclusion of Venkatesh et al. [38], in this study, effort expectation has no significant influence on college students’ intention to learn BIM technology (β = −0.035, *p* > 0.05). This result is surprising because it is inconsistent with most previous research conclusions. For example, the research of Li and Zhao [39] reflects that effort expectancy is expected to have a significant positive impact on the intention of continuing to use MOOC. VanDerSchaaf et al. [40] research also shows that effort expectancy and social influence are the key influences on college students’ intention to use information technology to obtain university services. However, the situation of this study is not without precedent. For instance, in the study of influencing factors of doctors’ adoption of electronic health records by Hossain et al. [41], it was concluded that efforts are expected to have no significant impact on doctors’ adoption of electronic health records. Andrews et al. [42] found that the effort is expected to have no significant impact on librarians’ adoption of AI and related technologies. This work [43] suffers the same limitations as social networking sites, and effort expectancy has no significant influence on behavior intention. In the study of Indian graduate students’ intention and obstacles using MOOCs by Mohan et al. [45], relevant conclusions have also been drawn. Efforts expectancy, social influence, and facilitating conditions have no statistically significant influence on the intention to use MOOCs. In the research of pharmaceutical students’ acceptance of LabSafety based on mobile devices by Ameri et al. [44], it was also found that effort expectancy has no significant influence on their intention to use. Further analysis shows that there might be two reasons for the insignificant influence of effort expectation on learning intention in this study: First, college students are generally younger (ranging from 19 to 25 years old). Therefore, they can easily accept the new technology, and it is less cumbersome to learn BIM technology since potential hardship in learning new technologies can not affect their intention to learn. Second, only basic software operations are being taught in many schools. Many complex functions have yet to be explored by students. Therefore, it is not clear what efforts are needed to learn BIM technology, and this issue has been mentioned by many students in questionnaire interviews. As shown in the scores of the performance expectancy items, the scores are mainly distributed between “general” and “basic agreement”, which is closer to “general”, and it can be seen that most students do not know much about BIM technology’s performance expectancy or perceived ease of use. In other words, students do not know much about the difficulty of BIM technology. They are only informed about BIM technology’s capabilities to obtain better job opportunities, but they do not know exactly how and what to learn. Therefore, some college students’ understanding of BIM technology is still insufficient and there is no significant statistical relationship between their expectation and intention to learn BIM technology.

## 6. Conclusions and Implications

Through sorting out and analyzing a large number of related literature works, this study uses UTAUT to construct the model of influencing factors of BIM technology learning intention and behavior from the perspective of students’ perceptions. A random sample questionnaire survey was conducted among AEC majors in four types of universities (research-oriented, applied research-oriented, application-oriented, and private research-oriented) in seven regions of China (northeast, north, east, south, central, northwest, and southwest), and the influencing factors and their relationships were explored. The results show that, in addition to effort expectancy, performance expectancy, social influence, and learning attitude have significant direct effects on learning intention and have significant indirect effects on learning behavior through learning intention; facilitating conditions and learning intention have significant direct effects on learning behavior. This study once again verified the effectiveness of UTAUT in the field of BIM technology learning and confirmed the significant influence of attitude on BIM technology learning intention and behavior.

### 6.1. Practical Implications

Based on the research conclusion, this study puts forward the following suggestions for further improving the BIM technology education for college students:

1. Given the significant positive influence of learning attitude on learning intention and behavior, the learning objectives of undergraduate students need to be clarified to improve the learning attitude towards BIM technology. Therefore, to make students’ learning objectives clear, the curriculum arrangement in colleges and universities should include basic knowledge of BIM and career planning. In this way, first-year students can overcome their fear of ignorance of BIM industry. Moreover, a well-established BIM curriculum may assist students in realizing the importance of BIM technology to the future of AEC industry.

2. Because of the significant positive influence of facilitating conditions on learning behavior, the BIM technology training for teachers should be strengthened to improve BIM technology operation and teaching ability. The more the teachers are capable of teaching BIM technology, the more support they can provide for the students. Thus, the improvement of teachers’ professional skills will be beneficial to BIM technology education. At present although, college teachers are primarily the young generation with scientific solid research abilities, their teaching ability is generally not enough due to the limitation of working time and experience [62]. Therefore, college teachers must carry out teaching skills training to improve their professional quality.

3. Given the significant positive influence of facilitating conditions on learning behavior, resources and support for learning BIM technology ought to be enhanced. The leading exporters of BIM talents, colleges, and universities should provide comprehensive support for the application of BIM technology from the aspects of system, human resources, funds, and policies [63]. In addition to ensuring the quality of BIM courses taught in schools, colleges, and universities, those organizations should encourage and support students to participate in various BIM competitions to improve undergraduate students’ BIM application ability. This suggestion may lead to continuously training qualified AEC graduates for the industry.

4. In view of the significant positive influence of social influence on learning intention and behavior, the promotion of BIM industry and the creation of BIM learning and application atmosphere are vital. The higher the industry adoption of BIM technology, the stronger the willingness of college students to learn. Hence, the government should provide policy and economic support to enterprises, encourage the construction industry to apply BIM technology, and actively promote BIM technology, creating a solid learning atmosphere for BIM technology educators and learners.

### 6.2. Future Work

This study adopts the UTAUT model, which has a very high degree of explanation for user intention and behavior. However, the model does not consider individual students’ learning abilities and methods. Future directions of work focus on adding constructivist learning theory and considering learning self-efficacy on this research basis. In addition, considering that too many questions in the online questionnaire may seem boring for respondents, the number of questionnaire items is relatively small. However, fewer items in each latitude may make deeper content impossible to explore. In the following research, each dimension can be further subdivided in detail, and additional items can be added to make the measurement results more accurate.

## Figures and Tables

**Table 1:** Research hypotheses.

Hypothesis Number	Research Hypothesis	Reference
H1	College students’ attitude toward BIM technology will positively affect their learning intention.	[42]
H2	College students’ performance expectancy of BIM technology will positively affect their learning intention.	[38,39,56]
H3	College students’ efforts expectancy of BIM technology will positively affect their learning intention.	[38,39,56]
H4	College students’ social influence on BIM technology will positively affect their learning intention.	[38,39,56]
H5	College students’ facilitating conditions of BIM technology will positively affect their learning behavior.	[38,50]
H6	College students’ behavioral intention toward BIM technology will positively affect their learning behavior.	[50]

**Table 2:** Sample school selection results.

Areas	Research-Oriented University	Applied Research-Oriented University	Application-Oriented University	Private Application-Oriented University
Central China	Wuhan University	China Three Gorges University	Zhengzhou University of Aeronautics	College of Science and Technology of China Three Gorges University
		Hunan University of Science and Technology		
		Changsha University of Science and Technology		
East China	Hefei University of Technology	Shandong Jianzhu University	Suzhou University of Science and Technology	Tianping College of Suzhou University of Science and Technology
				Anhui Xinhua University
North China	Beijing Jiaotong University	Shijiazhuang Tiedao University	Inner Mongolia University of Science and Technology	Shanxi Technology and Business University
	Central University of Finance and Economics			
Northeast China	Northeast Forestry University	Shenyang Jianzhu University	Dalian Ocean University	East University of Heilongjiang
			Liaoning University of Technology	Jilin University of Architecture and Technology
Northwest China	Chang’an University	Xi’an University of Architecture and Technology	HeXi University	Ningxia Institute of Science and Technology
SouthwestChina	Chongqing University	Southwest University Of Science And Technology	Leshan Normal University	Southwest Jiaotong University Hope College
		Kunming University of Science and Technology		
South China	South China University of Technology	Hainan University	Wuyi University	University of Sanya

**Table 3:** Descriptive statistics.

Topic Item	Option	Count Heads	Percentage of the Total Number of People
gender	Male	455	42%
	Female	635	58%
age	19 years or younger	58	5%
	19–21 years old	749	69%
	22–24 years old	280	26%
	Over 24 years old	3	0.3%
grade	Freshman	214	20%
	Sophomore	357	33%
	Junior	299	27%
	Senior	220	20%
specialized subject	Engineering management	553	50.7%
	Construction costs	247	22.7%
	Civil engineering	94	8.6%
	Architecture	60	5.5%
How do you know the BIM industry?	Understand	255	24%
	Common	232	21%
	Incomprehension	603	55%
Whether you have participated in BIM competitions or not?	Yes	188	17%
	No	902	83%
Whether the school offer BIM courses or not?	Yes	813	75%
	No	277	25%

**Table 4:** Results table of confirmatory factor analysis.

Variable	Topic Item	Factor Loading	CR	AVE
performance expectancy	PE1	0.808	0.911	0.773
	PE2	0.946		
	PE3	0.879		
effort expectancy	EE1	0.754	0.873	0.698
	EE2	0.881		
	EE3	0.865		
social influence	SI1	0.881	0.860	0.675
	SI2	0.888		
	SI3	0.678		
facilitating conditions	FC1	0.74	0.850	0.586
	FC2	0.823		
	FC3	0.749		
	FC4	0.747		
learning attitude	LA1	0.835	0.916	0.786
	LA2	0.883		
	LA3	0.938		
learning intention	LI1	0.86	0.866	0.683
	LI2	0.833		
	LI3	0.785		
learning behavior	LB1	0.897	0.891	0.803
	LB2	0.895		

**Table 5:** Table of distinctions.

	Performance Expectancy	Effort Expectancy	Social Influence	Facilitating Conditions	Learning Attitude	Learning Intention	Learning Behavior
performance expectancy	0.879						
effort expectancy	0.276 ***	0.835					
social influence	0.472 ***	0.368 ***	0.822				
facilitating conditions	0.507 ***	0.404 ***	0.537 ***	0.766			
learning attitude	0.389 ***	0.357 ***	0.420 ***	0.479 ***	0.887		
learning intention	0.424 ***	0.280 ***	0.464 ***	0.444 ***	0.763 ***	0.826	
learning behavior	0.091 **	0.380 ***	0.175 ***	0.306 ***	0.270 ***	0.293 ***	0.896

Note: ***, *p* < 0.001; **, *p* < 0.01; Diagonal line is SQRT(AVE).

**Table 6:** Structural model fitting index.

Index	CMIN/DF	GFI	AGFI	RMSEA	NFI	TLI	CFI
Text value	4.541	0.934	0.911	0.057	0.948	0.950	0.959
Recommended value	<5	>0.9	>0.9	<0.08	>0.9	>0.9	>0.9

**Table 7:** Total effect, direct effect, and indirect effect.

Variable	Effect Type	AT	SI	EE	PE	FC	LI
LI	Total	0.675 ***	0.144 **	−0.035	0.101 ***	-	-
	Direct	0.675 ***	0.144 **	−0.035	0.101 ***	-	-
	Indirect	-	-	-	-	-	-
LB	Total	0.137 ***	0.029 ***	−0.007	0.02 ***	0.212 ***	0.203 ***
	Direct	-	-	-	-	0.212 ***	0.203 ***
	Indirect	0.137 ***	0.029 ***	−0.007	0.02 ***	-	-

Note: ** means less than 0.001 < *p* < 0.005, and *** means less than *p* < 0.001.

## Data Availability

The data used to support the findings of this study are available from the corresponding author upon reasonable request.

## Appendix A. Measures

Performance expectancy:

PE1—Learning BIM technology is helpful for my studies.

PE2—Learning BIM technology is helpful for my career development.

PE3—Learning BIM technology can make me more competitive when I enter the workplace in the future.

Effort expectancy:

EE1—I think BIM software interface design is reasonable and easy to operate.

EE2—I think it’s easy to learn to use BIM software.

EE3—It’s easy for me to be proficient in using BIM software.

Social influence:

SI1—People who are important to me think I should learn BIM technology.

SI2—People who influence my behavior think I should learn BIM technology.

SI3—The promotion and popularization of BIM technology in the construction industry prompted me to learn BIM.

Facilitating conditions:

FC1—The school supports us to learn BIM technology.

FC2—The BIM instructor can give me useful suggestions.

FC3—I have the resources needed to learn BIM technology.

FC4—When I encounter difficulties in learning BIM technology, teachers/classmates can help me solve the difficulties.

Learning attitude:

LA1—It is worthwhile to learn BIM technology.

LA2—I am interested in BIM technology.

LA3—It’s fun to learn BIM technology.

Learning intention:

LI1—I want to continue learning BIM technology in the future.

LI2—I want to work related to BIM in the future.

LI3—I’d like to take part in the BIM competition.

Learning behavior:

LB1—I can operate related BIM software.

LB2—I am learning or have already learned BIM technology.
